# Treatment and Outcomes of Thrombolysis Related Hemorrhagic Transformation: A Multi-Center Study in China

**DOI:** 10.3389/fnagi.2022.847648

**Published:** 2022-04-07

**Authors:** Junfeng Liu, Yanan Wang, Jing Li, Shanshan Zhang, Qian Wu, Chenchen Wei, Ting Cui, Bo Wu, Joshua Z. Willey, Ming Liu

**Affiliations:** ^1^Department of Neurology, Center of Cerebrovascular Diseases, West China Hospital, Sichuan University, Chengdu, China; ^2^Department of Neurology, The First People’s Hospital of Ziyang, Ziyang, China; ^3^Department of Neurology, Mianyang Central Hospital, Mianyang, China; ^4^Department of Neurology, The Affiliated Hospital of Qingdao University, Qingdao, China; ^5^Department of Neurology, Columbia University Irving Medical Center, New York, NY, United States

**Keywords:** acute ischemic stroke, hemorrhagic transformation, thrombolysis, treatment, outcomes

## Abstract

**Objective:**

To investigate the current management of thrombolysis related hemorrhagic transformation (HT) in real-world practice, and whether these treatments would reduce the risk of 3-month death and hematoma expansion after HT.

**Methods:**

A multicenter retrospective study was performed in three comprehensive stroke centers in China (West China Hospital, The First People’s Hospital of Ziyang, and Mianyang Central Hospital) between January 1st 2012 and December 31th 2020. Participants were patients diagnosed with HT after intravenous thrombolytics on brain computed tomography (CT) within 36 h after stroke onset. The treatment after thrombolysis related HT included aggressive therapy (procoagulant, neurosurgical treatment) and dehydration therapy (mannitol or glycerin and fructose). The primary clinical outcome was 3-month death. The primary radiographic outcome was hematoma expansion, defined as a 33% increase in the hematoma volume using the (A × B × C)/2 method on follow-up imaging.

**Results:**

Of 538 patients with ischemic stroke receiving thrombolysis included during the study period, 94 patients (17.4%) were diagnosed with HT, 50% (47/94) of whom were symptomatic HT. The 3-month death was 31.5% (29/92), with two patients having been lost to follow up. A total of 68 patients (72.3%) had follow-up brain CT scans after HT detection for evaluating hematoma expansion, of whom 14.7% (10/68) had hematoma expansion. Among the 10 patients with hematoma expansion, 7 patients were from symptomatic HT group, and 3 patients were from the asymptomatic hematoma group. In regard to escalation in therapy, six patients received neurosurgical treatment and three patients had a fresh frozen plasma infusion. In addition, dehydration therapy was the most common management after HT diagnosis [87.2% (82 of 94)]. In the multivariable models, refusing any treatment after HT diagnosis was the sole factor associated with increased 3-month death (odds ratio, 13.6; 95% CI, 3.98–56.9) and hematoma expansion risk (odds ratio, 8.54; 95% CI, 1.33–70.1). In regard to the effects of aggressive therapy, a non-significant association of receiving hemostatic/neurosurgery therapy with a lower 3-month death and hematoma expansion risk was observed (all *P* > 0.05).

**Conclusion:**

Refusing any treatment after HT detection had a significant trend of increasing 3-month death and hematoma expansion risk after HT. Our finding of hematoma expansion among patients with asymptomatic HT in non-western populations suggests an opportunity for intervention. Very few patients after thrombolysis related HT diagnosis received procoagulant or neurosurgical therapies. Large multicenter studies enrolling diverse populations are needed to examine the efficacy of these therapies on different HT subtypes.

## Introduction

Hemorrhagic transformation (HT) is a common complication of ischemic stroke associated with higher mortality and morbidity, and its frequency and severity can be increased by thrombolysis, thrombectomy, and early anticoagulation (Álvarez-Sabín et al., 2013). HT is commonly classified into symptomatic HT and asymptomatic HT based on the development of neurological deterioration attributable to the hemorrhage. The Heidelberg bleeding classification (von Kummer et al., 2015) focuses on radiographic criteria, including hemorrhagic infarction (HI) type-1, HI-2, parenchymal hematoma (PH) type-1, PH-2, and remote intracerebral hemorrhage. However, in clinical practice, physicians prefer to use the European Cooperative Acute Stroke Study (ECASS) classification including HI-1, HI-2, PH-1, and PH-2 because of its simplicity and higher interrater agreement (Dowlatshahi et al., 2011; Seet and Rabinstein, 2012).

The general principles of HT management are similar to those used in treating spontaneous intracerebral hemorrhage such as treatment of elevated intracranial pressure, and prevention of hematoma expansion (Hemphill et al., 2015). For symptomatic HT after thrombolysis, anti-fibronolytics agents were suggested by the American Heart Association and the American Stroke Association (Yaghi et al., 2017) in all symptomatic HT. In addition, other agents for reversal of the coagulopathy may also be considered including cryoprecipitate to achieve a fibrinogen level of ≥150 mg/dL, platelet transfusion in those with thrombocytopenia (platelet count <100,000/μL), and Vitamin K/fresh-frozen plasma/prothrombin complex concentrate as an adjunctive therapy in those on warfarin treatment (Yaghi et al., 2017). Few studies have investigated the management after HT (Goldstein et al., 2010; Alderazi et al., 2014), all of which focused on thrombolysis-related symptomatic HT and were from western countries. Even fewer study (Yaghi et al., 2015) has examined the use and effectiveness in real-time clinical practice of recommended therapies in HT after thrombolysis, especially in Asian populations such as in China where the prevalence of HT may be higher due to the potential racial differences in coagulation-fibrinolysis factors and genetic polymorphism (Ueshima and Matsuo, 2002; Shen et al., 2007; Menon et al., 2012; Mehta et al., 2014). Given the higher prevalence and limited data on treatment, additional data is needed to guide further improvements in the quality of care.

Therefore, we performed a retrospective, real-world study in three comprehensive stroke centers in China to investigate: (1) the current management of HT after thrombolysis in the real-world practice; (2) and their effects on 3-month death and hematoma expansion after HT.

## Materials and Methods

### Study Participants

Ischemic stroke patients aged 18 years or older who were admitted within 6 h after stroke were retrospectively retrieved from three primary and comprehensive stroke centers in Sichuan province, China (West China Hospital, The First People’s Hospital of Ziyang, and Mianyang Central Hospital) during January 1st 2012–December 31th 2020. Participants were included in this study if they received intravenous thrombolysis with alteplase at 0.9 mg/kg or received thrombectomy after thrombolysis (i.e., bridging therapy). Patients diagnosed with HT on computed tomography (CT) scan within 36 h after onset were identified at each center. In all of the stroke centers a follow up CT was required within 24 h before starting anti-thrombotic therapy. Apart from this, additional CT was also performed based upon clinicians’ preference or with neurological deterioration during hospitalization.

Acute ischemic stroke was diagnosed according to the World Health Organization criteria, and further confirmed by a CT or magnetic resonance imaging (MRI) scan. The study was approved by the Biomedical Research Ethics Committee of each center.

### Demographic, Vascular Risk Factor, and Other Clinical Variables Collection

We collected demographic and medical history including age, sex, hypertension, diabetes mellitus, atrial fibrillation, hyperlipidemia, smoking, and alcohol consumption. Treatment before thrombolysis including antiplatelet or anticoagulant use was also collected. NIHSS score on admission (Brott et al., 1989) was assessed by an experienced neurologist. Determination of ischemic stroke etiology was according to the Trial of Org 10172 in Acute Stroke Treatment (TOAST) criteria: large-artery atherosclerosis (LAA); small-artery occlusion (SAO); cardioembolism (CE); stroke of other determined cause (OC); and stroke of undetermined cause (UND) (Adams et al., 1993). Pretreatment blood glucose level, initial platelet count and fibrinogen level after HT diagnosis were also retrieved from the medical records.

If patients were found to have symptomatic HT within 24 h after thrombolysis, the anti-thrombotic therapy will not be initiated. The time of restarting the anti-thrombotic therapy, and other treatments including aggressive therapy (procoagulant or neurosurgical treatment) and dehydration therapy (mannitol or glycerin and fructose) were based on patients’ conditions and clinicians’ preference. The standard protocol of treating HT after thrombolysis was not available in clinical practice, especially for patients with asymptomatic HT.

The treatment after HT including aggressive therapy (procoagulant or neurosurgical treatment) and dehydration therapy (mannitol or glycerin and fructose) were retrieved from the medical records. In addition, anti-thrombotic therapy including oral antiplatelets (such as aspirin and clopidogrel) and anticoagulation (such as warfarin and novel oral anticoagulant) after HT was also collected from the records. Procoagulant therapies consist of using fresh frozen plasma, cryoprecipitate, vitamin K, platelet transfusion, recombinant factor VIIa, or aminocaproic acid. Dehydration therapy was used when signs of brain edema or brain hernia were observed on brain imaging. The dose and duration of mannitol or glycerin and fructose was determined at the discretion of the responsible neurologists. Neurosurgical treatment included surgical decompressive craniotomy or hematoma evacuation. The primary clinical outcome was 3-month death, based on medical records or the interview with a relative by telephone at 3 months after stroke onset. The secondary clinical outcome was the combined outcome of death/disability at 3 months, defined as a score of 3–6 on the modified Rankin Scale (mRS) score (De Haan et al., 1995). The primary radiographic outcome was hematoma expansion, defined as a 33% increase in the hematoma volume using the (A × B × C)/2 method on follow-up imaging (Anderson et al., 2008). Not all the patients with a HT diagnosis had follow-up scans to evaluate hematoma expansion. The repeated imaging was performed if the patients had acute neurologic decline or based on the physician’s preference.

### Definition of Hemorrhagic Transformation

All patients underwent brain imaging including non-contrast CT on admission, and also had a follow-up CT within 36 h after stroke onset. Any hemorrhage found on the follow-up CT brain scans after admission but not detected on initial scans was defined as HT. Symptomatic HT and asymptomatic HT were diagnosed according to the definition of the National Institute of Neurological Disease and Stroke study: any neurological worsening within 36 h of tissue plasminogen activator administration that is attributed to HT verified by CT or MRI scan (The National Institute of Neurological Disorders and Stroke rt-Pa Stroke Study Group, 1995). Regarding the radiological classification, HT was classified as HI-1, HI-2, and PH-1, PH-2 based on the European Cooperative Acute Stroke Study III definition (de Los Ríos la Rosa et al., 2012).

### Statistical Analysis

Descriptive statistics for the baseline demographic data and vascular risk factors were presented. A *t*-test, analysis of variance, the Chi-square test and fisher exact test were used to compare continuous and categorical variables between groups accordingly. The variables showed a univariate relationship with 3-month death, 3-month death/disability, and hematoma expansion (*P* < 0.1) were further analyzed in multivariate logistic regression models. Two-sided values of *P* < 0.05 were considered statistically significant. All statistical analyses were performed using the R statistical programming environment (version 3.4.1.^1^).

## Results

### Baseline Characteristics

**FIGURE 1:**
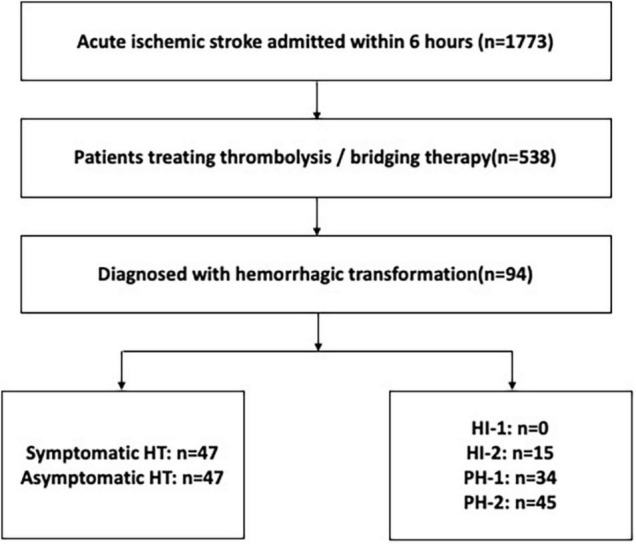
The flowchart of patients. HT, hemorrhagic transformation; HI, hemorrhagic infarction; PH, parenchymal hematoma.

Between January 1st 2012 and December 31th 2020, a total of 481 patients were enrolled from West China Hospital. From June 1st 2017 and March 31th 2019, a total of 22 and 35 patients were included from Mianyang Central Hospital and The First People’s Hospital of Ziyang, respectively. Among the 538 patients with thrombolysis or bridging therapy, 94 ischemic stroke patients with HT after thrombolysis were finally included in the present study. Figure 1 shows the flow of patients in the study. A total of 94 patients (17.5%) were found to have HT on CT within 36 h after stroke onset, of whom 47 (50%) had symptomatic HT and 47 (50%) had asymptomatic HT. Among the 94 patients with HT, the imaging classification was 15 (15.9%) with HI-2, 34 (36.2%) with PH-1, and 45 (47.9%) with PH-2. In the final analysis, 94 patients with HT were included (Figure 1) with 55 patients receiving thrombolytics only and 39 patients having thrombectomy after intravenous thrombolytics (i.e., bridging therapy). The baseline characteristics of the 94 patients with thrombolysis related HT are summarized in Table 1. The mean age was 69 years, and 48 patients (51.1%) were male. The median time from stroke onset to thrombolysis was 3.00 h (IQR 2.0–4.0 h). The median time from stroke onset to any HT detection was 23.9 h (IQR 11.5–27.5 h). The detection time after stroke onset of symptomatic HT was 14.1 h (IQR 11.0–24.5 h), and 25.6 h (IQR 20.1–28.1 h) for asymptomatic HT.

**TABLE 1:** The baseline characteristics of patients with hemorrhagic transformation.

Characteristics	All (*n* = 94)
Onset to treatment time (hours), median (IQR)	3.00 (2.0, 4.0)
Onset to HT detection time (hours), median (IQR)	23.9 (11.5, 27.5)
Age (years), mean (SD)	68.8 (15.1)
Male, *n* (%)	48 (51.1)
Hypertension, *n* (%)	49 (52.1)
SBP (mmHg), mean (SD)	143.9 (28.1)
DBP (mmHg), mean (SD)	80.8 (13.7)
Diabetes mellitus, *n* (%)	21 (22.3)
Fasting glucose (mmol/L), mean (SD)	8.7 (3.3)
Platelet count on admission, mean (SD)	176.1 (52.1)
Atrial fibrillation, *n* (%)	42 (44.7)
Smoking, *n* (%)	27 (28.7)
Drinking, *n* (%)	26 (27.7)
Previous antiplatelet, *n* (%)	4 (4.3)
Previous anticoagulation, *n* (%)	6 (6.4)
Warfarin, *n* (%)	4 (4.3)
Novel oral anticoagulant, *n* (%)	2 (2.1)
NIHSS on admission, median (IQR)	16.00 (12.00, 19.00)
TOAST classification	
Large-artery atherosclerosis, *n* (%)	28 (29.8)
Small-artery occlusion, *n* (%)	0 (0.0)
Cardioembolic, *n* (%)	46 (48.9)
Undetermined etiology, *n* (%)	17 (18.1)
Other etiology, *n* (%)	3 (3.2)
Reperfusion treatment	
Only thrombolysis, *n* (%)	55 (58.5)
Bridging therapy, *n* (%)	39 (41.5)
Symptomatic hemorrhagic transformation, *n* (%)	47 (50.0)
ECASS classification	
Hemorrhagic infarction (HI)-1, *n* (%)	0 (0)
Hemorrhagic infarction (HI)-2, *n* (%)	15 (16.0)
Parenchymal hematoma (PH)-1, *n* (%)	34 (36.2)
Parenchymal hematoma (PH)-2, *n* (%)	45 (47.9)
Location of infarcts	
Anterior circulation, *n* (%)	82 (87.2)
Posterior circulation, *n* (%)	9 (9.6)
Anterior + posterior circulation, *n* (%)	3 (3.2)

*TOAST classification, Trial of Org 10172 in Acute Stroke Treatment classification; SBP, systolic blood pressure; DBP, diastolic blood pressure; HT, hemorrhagic transformation.*

### Evolution and Outcomes of Hemorrhagic Transformation

Among the 47 patients with initially asymptomatic HT, 1 patient (2.1%) became symptomatic at 10.8 h after HT detection. A total of 68 patients (72.3%) had follow-up brain CT scans after HT detection for evaluating hematoma expansion. In total, the frequency of hematoma expansion was 14.7% (10/68), and the median time from HT detection to hematoma expansion was 3.5 days (range: 1.2–5.8 days). Among the 10 patients with hematoma expansion, 7 patients were from symptomatic HT group, and 3 patients were from the asymptomatic hematoma group.

Two patients were lost to follow up at 3 months after stroke. The 3-month death and death/disability were 31.5% (29/92) and 59.8% (55/92), respectively.

### Real-World Treatment Practice of Hemorrhagic Transformation

A total of 40 patients (42.6%) were suggested to have neurosurgery by surgeons, but 34 of them refused to have surgery. Therefore, the neurosurgery was performed in only six patients, five of them from the symptomatic HT group, and the other one from asymptomatic HT group (PH1 type). The fibrinogen levels after HT detection were available in 38 patients (40.4%), 8 of whom had hypofibrinogenemia (fibrinogen level, <2.0 g/L), and only 3 patients received fresh frozen plasma infusion. Meanwhile, 82 patients (82/94, 87.2%) were treated with dehydration therapy, 50 patients of whom used osmotic drugs more than 7 days. In regard to different osmotic drugs, 43 patients only used mannitol, 2 patients with glycerol and fructose only, and 36 patients used both of them. The median dose of mannitol was 75 (50–100) g/day, and the median dose of glycerol and fructose was 250 (250–500) ml/day. In addition, 29 families (30.1%) refused to receive any treatment and asked for discharging home after HT detection, 24 of whom were with symptomatic HT.

In regard to the anti-thrombotic therapy, among the 48 patients (51.1%) diagnosed with HT within 24 h after onset before starting anti-thrombotics, only three patients still initiated because of stenting. The remaining 46 patients who had HT during 24–36 h after onset, 15 patients of whom continued anti-thrombotic treatment, and 31 patients (67.4%) discontinued anti-thrombotics, 12 of whom restarted during hospitalization. A total of 77 patients were alive at discharge, 30 patients were prescribed with anti-thrombotics at discharge, 23 of whom were with antiplatelets, and 7 patients with anticoagulants. Among the 47 patients without anti-thrombotics at discharge, 17 patients were found to be dead at 3 months.

### Treatment Effects on the Outcomes of Hemorrhagic Transformation

#### Three-Month Death and Death/Disability

In the univariate analysis, the receipt of receiving dehydration therapy and refusing any treatment were the statistically significant factors associated with a higher 3-month death (all *P* ≤ 0.02, Table 2). However, there was a non-significant suggestion of lower 3-month death in patients receiving procoagulant therapy/neurosurgery for HT (*P* = 0.68). The differences between patients who died and those who survived at 3 months were summarized in Table 2. After adjusting for smoking, previous anticoagulation, dehydration therapy, refusing any treatment and symptomatic HT, the only variable associated with increased 3-month death was refusing any treatment after HT detection (odds ratio, 13.6; 95% CI, 3.98–56.9; *P* < 0.001).

**TABLE 2:** Univariate analysis of predictors of 3-month death and hematoma expansion.

	3-month prognosis cohort (*n* = 92)			Hemorrhagic transformation cohort with follow-up scans (*n* = 68)		
		
Variables	3-month alive (*n* = 63)	3-month death (*n* = 29)	*P*	No hematoma expansion (*n* = 58)	Hematoma expansion (*n* = 10)	*P*
Onset to treatment time (hours), median (IQR)	3.0 (2.0, 3.2)	3.0 (2.0, 4.0)	0.38	3.0 (2.0, 3.1)	3.5 (2.3, 4.0)	0.48
Onset to HT detection time (hours), median (IQR)	24.5 (12.2, 27.7)	17.2 (10.9, 25.8)	0.21	24.4 (11.2, 27.9)	23.7 (20.7, 25.8)	0.96
Age (years), mean (SD)	67.4 (16.0)	71.5 (12.7)	0.23	67.2 (16.3)	73.8 (7.5)	0.22
Male, *n* (%)	35 (55.6)	11 (37.9)	0.18	33 (56.9)	5 (50.0)	0.74
Hypertension, *n* (%)	35 (55.6)	13 (44.8)	0.38	29 (50.0)	7 (70.0)	0.31
SBP (mmHg), mean (SD)	145.1 (28.8)	142.9 (27.4)	0.74	142.3 (25.4)	152.7 (31.0)	0.25
DBP (mmHg), mean (SD)	80.8 (13.8)	81.3 (14.0)	0.88	81.1 (13.7)	81.3 (10.9)	0.97
Diabetes mellitus, *n* (%)	11 (17.5)	9 (31.0)	0.18	7 (12.1)	3 (30.0)	0.16
Fasting glucose (mmol/L), mean (SD)	8.5 (3.5)	9.00 (2.8)	0.53	8.2 (3.0)	8.2 (2.00)	0.96
Platelet count on admission, mean (SD)	176.9 (51.0)	171.1 (53.5)	0.62	176.1 (51.9)	157.9 (38.0)	0.29
Atrial fibrillation, *n* (%)	26 (41.3)	16 (55.2)	0.26	23 (39.7)	6 (60.0)	0.31
Smoking, *n* (%)	23 (36.5)	4 (13.8)	0.03	19 (32.8)	2 (20.0)	0.71
Drinking, *n* (%)	21 (33.3)	5 (17.2)	0.14	18 (31.0)	3 (30.0)	0.95
Previous antiplatelet, *n* (%)	3 (4.8)	1 (3.4)	0.77	2 (3.4)	0 (0.0)	0.55
Previous anticoagulation, *n* (%)	1 (1.6)	5 (17.2)	0.01	3 (5.2)	1 (10.0)	0.48
NIHSS on admission, median (IQR)	16.00 (11.50, 18.00)	15.00 (13.00, 21.00)	0.26	14.0 (10.3, 18.8)	21.5 (17.3, 22.8)	0.004
TOAST classification			0.31			0.87
Large-artery atherosclerosis, *n* (%)	19 (30.2)	7 (24.1)		20 (34.5)	3 (30.0)	
Small-artery occlusion, *n* (%)	0 (0)	0 (0)		0 (0)	0 (0)	
Cardioembolic, *n* (%)	30 (50.8)	14 (48.3)		26 (44.8)	5 (50.0)	
Undetermined etiology, *n* (%)	3 (4.8)	0 (0)		3 (5.2)	0 (0)	
Other etiology, *n* (%)	9 (14.3)	8 (27.6)		9 (15.5)	2 (20.0)	
**Type of treatment**						
Dehydration therapy, *n* (%)	51 (81.0)	29 (100.0)	0.02	48 (82.8)	10 (100.0)	0.34
Procoagulant therapy or neurosurgery, *n* (%)	6 (9.5)	2 (6.9)	0.68	4 (6.9)	0 (0.0)	0.39
Procoagulant therapy, *n* (%)	2 (3.2)	1 (3.4)	0.95	3 (5.2)	0 (0.0)	0.46
Neurosurgery, *n* (%)	5 (7.9)	1 (3.4)	0.67	2 (3.4)	0 (0.0)	0.55
Refusing treatment	9 (14.3)	20 (69.0)	<0.001	7 (12.1)	4 (40.0)	0.049
Symptomatic hemorrhagic transformation, *n* (%)	26 (41.3)	21 (72.4)	0.007	22 (37.9)	7 (70.0)	0.09
ECASS classification			0.21			0.39
Hemorrhagic infarction (HI)-1, *n* (%)	0 (0)	0 (0)		0 (0)	0 (0)	
Hemorrhagic infarction (HI)-2, *n* (%)	11 (17.5)	2 (6.9)		11 (19.0)	0 (0.0)	
Parenchymal hematoma (PH)-1, *n* (%)	25 (39.7)	9 (31.0)		23 (39.7)	5 (50.0)	
Parenchymal hematoma (PH)-2, *n* (%)	27 (42.9)	18 (62.1)		24 (41.4)	5 (50.0)	
Location of infarcts			0.50			0.76
Anterior circulation, *n* (%)	55 (87.3)	25 (86.2)		50 (86.2)	9 (90.0)	
Posterior circulation, *n* (%)	5 (7.9)	4 (13.8)		5 (8.6)	1 (10.0)	
Anterior + posterior circulation, *n* (%)	3 (4.8)	0 (0.0)		3 (5.2)	0 (0.0)	

*TOAST classification, Trial of Org 10172 in Acute Stroke Treatment classification; SBP, systolic blood pressure; DBP, diastolic blood pressure; HT, hemorrhagic transformation.*

The differences between patients with 3-month death/disability and those without were summarized in Supplementary Table 1. Regarding to the treatment after HT, refusing any treatment was significantly associated with a higher risk of 3-month death/disability (*P* = 0.01) in the univariate analysis. However, the significant association was disappeared after adjusting the confounders.

#### Hematoma Expansion

The only significant variable related to higher risk of hematoma expansion after HT detection was NIHSS score on admission (median: 21.5 vs. 14.0, *P* = 0.004). In regard to the treatment, there was a marginally significant suggestion of increasing the risk of hematoma expansion in patients refusing any treatment (*P* = 0.05, Table 2). In addition, a non-significant association of receiving hemostatic/neurosurgery therapy with a lower hematoma expansion risk was observed (Table 2, all *P* > 0.05). After adjustment for confounders, higher initial NIHSS score (odds ratio, 1.3; 95% CI, 1.11–1.60; *P* = 0.005) and refusing any treatment (odds ratio, 8.54; 95% CI, 1.34–70.1; *P* = 0.03) were significantly related to a higher hematoma expansion risk after HT diagnosis.

In the end, we investigated the characteristics and outcomes of the eight patients who received neurosurgery or fresh frozen plasma infusion (Table 3). Two patients (22.7%) were found to be dead at 3 months after stroke onset. Four patients had the follow up scans for evaluating hematoma expansion, none of whom were found to have hematoma expansion during hospitalization.

**TABLE 3:** The characteristics and outcomes of the patients received surgery or fresh frozen plasma (*n* = 8).

Patient age, years/sex	Reperfusion intervention	Therapy	HT type	Fibrinogen level after HT diagnosis	Hematoma expansion	3-month mortality
77/F	Bridging therapy	Surgical decompressive craniotomy, fresh frozen plasma	Asymptomatic HT, PH1	1.77	NO	NO
46/M	Bridging therapy	Surgical decompressive craniotomy	Symptomatic HT, PH2	2.44	Not available	NO
69/F	Bridging therapy	Surgical decompressive craniotomy	Symptomatic HT, PH2	2.28	Not available	NO
69/M	Thrombolysis	Fresh frozen plasma	Symptomatic HT, PH2	1.92	NO	NO
56/M	Thrombolysis	Surgical decompressive craniotomy	Symptomatic HT, PH1	Not available	Not available	NO
42/M	Thrombolysis	Fresh frozen plasma	Symptomatic HT, PH2	1.97	NO	Yes
56/M	Bridging therapy	Surgical decompressive craniotomy	Symptomatic HT, PH1	2.34	Not available	Yes
48/M	Thrombolysis	Surgical decompressive craniotomy	Symptomatic HT, PH1	2.83	NO	NO

*F, female; M, male; HT, hemorrhagic transformation; HI, hemorrhagic infarction; PH, parenchymal hematoma.*

## Discussion

To our knowledge, this is the first study to report current treatments after HT in China, a region of the world where there are reported higher rates of HT in comparison to other countries (Ueshima and Matsuo, 2002; Shen et al., 2007; Menon et al., 2012; Mehta et al., 2014), and which we also found. Overall, we found the most common management after HT was dehydration therapy. Refusing any treatment after HT detection had a significant trend of increasing 3-month mortality and hematoma expansion risk (all *P* ≤ 0.03). Moreover, very few patients received aggressive therapy including procoagulant or neurosurgical treatment.

The high proportion (29/94; 30.1%) of refusing treatment and low proportion (8/94; 8.5%) of receiving procoagulant or neurosurgical treatment, could be a reflection of perceived futility by providers of these treatments, scant guidelines for non-western populations, or other local practice variations. Even among patients with symptomatic HT, the frequency of receiving procoagulant therapies or surgery is much lower than a study from the United States reported (14.9 vs. 38.2%) (Yaghi et al., 2015). Given this heterogeneity in treatment, significant opportunities for improvement in management after HT remain in regions such as China, and further research is needed as to the sources of this variability in order to improve outcomes after HT, and overall improve treatment rates for HT if the prognosis is understood not to be poor.

The current aggressive intervention including reversal agents of coagulopathy and neurosurgery has not been demonstrated to provide benefit in all HT. In our cohort, three patients with hypofibrinogenemia had fresh frozen plasma infusion, and its efficacy and safety has not been investigated yet. Future studies with large sample size may provide more evidence about this. In the study, we also reported a non-statistically significant association of neurosurgical treatment with a lower 3-month mortality and hematoma expansion risk, which is consistent with a previous study (Yaghi et al., 2015). Six of our patients received surgery after HT detection, none of whom had hematoma expansion and only one died at 3 months after stroke. It suggested that surgical intervention may be beneficial in a small proportion of patients with HT, similar to the results from spontaneous intracerebral hemorrhage (Hemphill et al., 2015).

In our cohort, 40 patients were suggested to have neurosurgery after HT diagnosis in our study, but 85% (36/40) families of these patients refused neurosurgery, and asked for non-invasive treatment. All the above suggested that treatment of HT, especially invasive treatment in China were under-utilized and need to improve compared to other geographic locations. In addition, there were 29 patients’ families who withdrew any treatment and indicated they wanted a discharge to home after HT detection. Several explanations may help us to understand the high proportion of refusing treatment and asking a discharge to home. First, in China, home has a special cultural meaning and dying at home is seen as a way of continuing bonds with one’s ancestors (Tang, 2000). A total of 82–92% of patients with severe conditions choose home deaths in mainland China (Cai et al., 2017; Dong et al., 2019). Second, the public health care in China cannot cover all the medical cost. The reimbursement rate was ranging from 14.2% in the lowest income group to 10.7% in the highest income group (Yip et al., 2019). Some families will chose to give up due to their financial condition.

In total, the frequency of hematoma expansion was 14.7% in the study, which suggests a potential window of opportunity for therapy and preventing ongoing bleeding. Among the 47 patients with symptomatic HT, only 29 patients had follow-up scans for evaluating hematoma expansion, 24.1% (7/29) of whom showed evidence of hematoma expansion, and a similar frequency has been reported in patients with thrombolysis related symptomatic HT (Goldstein et al., 2010; Yaghi et al., 2015). Apart from that, our finding that three of patients with asymptomatic HT also experienced hematoma expansion and one of them became symptomatic HT, suggests a necessity of identifying which patients may expand and therefore require treatment of asymptomatic bleeding, especially those with high risk of expansion. Large multicenter studies are needed to generate an algorithm to diagnose those patients who may have a high risk of evolution after HT detection in the future.

Among the 94 patients with HT in our study, 46 patients (48.9%) were cardioembolic stroke, but only 6 patients had anticoagulation before stroke (Table 1). The underusage of anticoagulation in China has also been previously reported by us (Liu et al., 2020). Cardioembolism has been reported to have the highest frequency of HT (Hakim et al., 1983), particularly when cardioembolism results in major arterial occlusion and failure of collateral flow. Moreover, HT is exceptional rare in cases of small vessel occlusion and lacunar stroke (Paciaroni et al., 2008; England et al., 2010), in consistent with our data that none of the 94 patients with HT were small-vessel occlusion stroke.

There are several potential limitations in our study. First, the study only included patients with thrombolysis from three stroke centers in Sichuan province, which is located in southwest of China, with a relatively lower quality of stroke care than the eastern and southern coasts in China (Wu et al., 2019). These factors may limit generalizability of our conclusions to some extent. Second, the small number of patients with neurosurgery or procoagulant therapy limited the efficiency of examining the effect of these treatment on outcomes. Third, not all patients with a HT diagnosis had follow-up scans to evaluate hematoma expansion, and the timing of follow-up CT was not standardized. In the study, there were 26 patients without follow-up scans after HT detection, 23 patients of whom cannot tolerate the radiological examination, and 3 patients discharged to home before examination. Therefore, the frequency of hematoma expansion may be not accurate. Future studies with follow-up scans performed systematically would provide more information. Last, we do not have the data of blood pressure within the first 24 h after thrombolysis, which may be related to the risk of hematoma expansion. The Enhanced Control of Hypertension and Thrombolysis Stroke Study (ENCHANTED) (Anderson et al., 2019) reported although intensive BP lowering (intensive target SBP to 130–140 mmHg within 1 h) was safe, the observed reduction in HT did not lead to improved clinical outcome compared with guideline treatment (SBP < 180 mmHg) (Powers et al., 2019). Whether intensive blood pressure lowering in patients with HT could reduce hematoma expansion needs further studies. Despite all that, our study is the first multicenter study in China, to our knowledge, that investigated real-world therapies used by comprehensive stroke centers after HT diagnosis in patients with thrombolysis.

## Conclusion

In the study, refusing any treatment after HT detection had a significant trend of increasing 3-month death and hematoma expansion risk after HT. Our finding of hematoma expansion among patients with asymptomatic HT in non-western populations suggests an opportunity for intervention. Very few patients after thrombolysis related HT diagnosis received procoagulant or neurosurgical therapies. There is significant room for improvement in management of HT in China. Large multicenter studies enrolling diverse populations are needed to examine the efficacy of these therapies on different HT subtypes.

## Data Availability Statement

The data that support the results of this study are available from the corresponding authors on reasonable request, without undue reservation.

## Ethics Statement

The studies involving human participants were reviewed and approved by the Ethics Committee of West China Hospital, Sichuan University [2016(339)]. Written informed consent for participation was not required for this study in accordance with the national legislation and the institutional requirements.

## Author Contributions

JfL drafted the manuscript and analyzed the data. YW, JL, SZ, QW, CW, and TC collected the data. BW and JW revised the manuscript. ML designed and supervised the research. All authors contributed to the article and approved the submitted version.

## Conflict of Interest

The authors declare that the research was conducted in the absence of any commercial or financial relationships that could be construed as a potential conflict of interest.

## Publisher’s Note

All claims expressed in this article are solely those of the authors and do not necessarily represent those of their affiliated organizations, or those of the publisher, the editors and the reviewers. Any product that may be evaluated in this article, or claim that may be made by its manufacturer, is not guaranteed or endorsed by the publisher.

## Footnotes

1
http://www.R-project.org
