# Supplementary material for: Treatment and Outcomes of Thrombolysis Related Hemorrhagic Transformation: A Multi-Center Study in China
Source: Front Aging Neurosci. 2022 Apr 7;14:847648. doi: 10.3389/fnagi.2022.847648 (PMC9021791; doi:10.3389/fnagi.2022.847648)
Supplement: Supplementary file 1 [file Table_1.DOCX]

**Supplementary Table I. Univariate analysis of predictors of 3-month death/disability**

|  | **3-month prognosis cohort(n=92)** | | |
| --- | --- | --- | --- |
| **Variables** | **3-month good outcome**  **(mRS score<3; n=37)** | **3-month death/disability**  **(mRS score 3-6; n=55)** | **P** |
| Onset to treatment time (hours), median (IQR) | 3.00 [2.00, 3.00] | 3.00 [2.00, 4.00] | 0.346 |
| Onset to HT detection time (hours), median (IQR) | 25.55 [17.92, 27.70] | 19.90 [10.90, 25.98] | 0.031 |
| Age (years), mean (SD) | 62.97 (17.46) | 72.55 (12.00) | 0.002 |
| Male, n (%) | 23 (62.2) | 23 (41.8) | 0.088 |
| Hypertension, n (%) | 17 (45.9) | 31 (56.4) | 0.396 |
| SBP (mmHg), mean (SD) | 133.35 (24.43) | 151.80 (28.42) | 0.002 |
| DBP (mmHg), mean (SD) | 78.27 (12.12) | 82.75 (14.50) | 0.125 |
| Diabetes mellitus, n (%) | 8 (21.6) | 12 (21.8) | 0.982 |
| Fasting glucose (mmol/L), mean (SD) | 8.90 (4.07) | 8.54 (2.70) | 0.618 |
| Platelet count on admission, mean (SD) | 180.11 (53.03) | 171.73 (50.79) | 0.448 |
| Atrial fibrillation, n (%) | 12 (32.4) | 30 (54.5) | 0.054 |
| Smoking, n (%) | 16 (43.2) | 11 (20.0) | 0.021 |
| Drinking, n (%) | 16 (43.2) | 10 (18.2) | 0.017 |
| Previous antiplatelet, n (%) | 2 (5.4) | 2 (3.6) | 0.683 |
| Previous anticoagulation, n (%) | 0 (0.0) | 6 (10.9) | 0.078 |
| NIHSS on admission, median (IQR) | 14.00 [9.00, 19.00] | 16.00 [13.00, 19.50] | 0.083 |
| Toast classification |  |  | 0.129 |
| Large-artery atherosclerosis, n (%) | 12 (32.4) | 14 (25.5) |  |
| Small-artery occlusion, n (%) | 0(0) | 0(0) |  |
| Cardioembolic, n (%) | 17 (45.9) | 29 (52.7) |  |
| Undetermined etiology, n (%) | 3 (8.1) | 0 (0.0) |  |
| Other etiology, n (%) | 5 (13.5) | 12 (21.8) |  |
| **Type of treatment** |  |  |  |
| Dehydration therapy, n (%) | 29 (78.4) | 51 (92.7) | 0.06 |
| Procoagulant therapy or neurosurgery, n (%) | 4 (10.8) | 4 (7.3) | 0.71 |
| Procoagulant therapy, n (%) | 2 (5.4) | 1 (1.8) | 0.342 |
| Neurosurgery, n (%) | 3(8.1) | 3 (5.5) | 0.613 |
| Refusing treatment | 6 (16.2) | 23 (41.8) | 0.012 |
| Symptomatic hemorrhagic transformation, n (%) | 12 (32.4) | 35 (63.6) | 0.005 |
| **ECASS classification** |  |  | 0.363 |
| Hemorrhagic Infarction (HI)-1, n (%) | 0(0) | 0(0) |  |
| Hemorrhagic Infarction (HI)-2, n (%) | 7 (18.9) | 6 (10.9) |  |
| Parenchymal hematoma (PH)-1, n (%) | 15 (40.5) | 19 (34.5) |  |
| Parenchymal hematoma (PH)-2, n (%) | 15 (40.5) | 30 (54.5) |  |
| **Location of infarcts** |  |  | 0.08 |
| Anterior circulation, n (%) | 30 (81.1) | 50 (90.9) |  |
| Posterior circulation, n (%) | 4 (10.8) | 5 (9.1) |  |
| Anterior+ Posterior circulation, n (%) | 3 (8.1) | 0 (0.0) |  |

Toast classification, Trial of Org 10172 in Acute Stroke Treatment classification; SBP, systolic blood pressure; DBP, diastolic blood pressure; HT, hemorrhagic transformation
